# *In-vitro* Pro Apoptotic Effect of Crude Saponin from *Ophiocoma erinaceus* against Cervical Cancer

**Published:** 2017

**Authors:** Elaheh Amini, Mohammad Nabiuni, Javad Baharara, Kazem Parivar, Javad Asili

**Affiliations:** a *Department of Animal Biology, Faculty of Biological Sciences, Kharazmi University, Tehran, Iran.*; b *Department of Cell and Developmental Biology, Kharazmi University, Tehran, Iran. *; c *Department of Biology, Research Center for Applied Biology, Mashhad Branch, Islamic Azad University, Mashhad, Iran. *; d *Department of Animal Biology, School of Basic Science, Science and Research Branch, Islamic Azad University, Tehran, Iran. *; e *Department of Pharmacognosy, School of Pharmacy, Biotechnology Research Center, Mashhad University of Medical Sciences, Mashhad, Iran.*

**Keywords:** Ophiocoma erinaceus, echinoderm, anticancer, cervical cancer, pro-apoptotic, intrinsic pathway

## Abstract

*Ophiocoma erinaceus* Muller &Troschel (Ophiocomidae) is part of the extensive group of echinoderm that contains bioactive metabolites. As the anti cancer potential of brittle star saponin has not been reported against cervical cancer, the present study was conducted to evaluate the anticancer effect of extracted crude saponin. Saponin extraction was conducted using conventional method such as froth test, TLC, FTIR and erythrolysis assay. The Hela-S3 cervical carcinoma and HNCF-PI52 normal cells were treated with different concentrations of saponin fraction for 24 and 48 h. The cytotoxicity was examined by MTT, DAPI, AO/PI, Annexin V-FITC and flow cytometry. In addition, the apoptotic induced pathway was studied using caspase assay, evaluation of ROS generation and Bcl-2 mRNA level. Crude saponin showed cytotoxic properties in Hela-S3 cells (IC_50_of 23.4 µg/mL) without significant impact against normal cells. In addition, the crude saponin increased sub-G1 peak in flow cytometry histogram of treated cells, ROS generation and caspase-3 and -9 activity (IC_50_ of 11.10, 11.27 µg/mL). The dose dependent down regulation of Bcl-2 in treated cells demonstrated that saponin fraction can trigger intrinsic apoptotic pathway in cancer cells. This study provides valuable information about the apoptotic inducing effect of saponin fraction, which can offer new insights into the anticancer potential of saponin as a promising candidate against human cervical carcinoma.

## Introduction

Ophiuroids have received less attention in biomedicine due to insufficient knowledge concerning how to deal with the presence of therapeutic metabolites (1). The existence of bioactive substances, such as steroids, naphtaquinone, terpens, phenylpropanoid and cerebrosides, have been demonstrated in these marine invertebrates (2). 

Saponins are steroidal terpenoids or sometimes alkaloid glycosides with several health benefits and a wide spectrum of pharmacological properties (3). The traditional application of saponins involves their usage as fish poison, detergents, food additives, surfactants for increment generation in milk and wool in ruminant production (4). The anticancer effect of saponins has been improved their pharmacological application in therapeutic fields(5).

Cervical cancer is one of the most prevalent cancers affecting women with a high rate of mortality (6). In spite of the great advances in cancer therapeutic methods, cervical cancer still represents a life threatening disorder for women with the crucial challenge being poor prognosis and lack of efficient standard chemotherapy approaches (7). The main therapeutic modalities for cervical cancer are surgery, radiation therapy and chemotherapy (8). 

However, apoptotic resistance is regarded as a major problem in response to conventional therapeutic methods and a substantial culprit for anticancer therapy failure (9). Trigger of apoptosis or the suppression of cancer cell growth is attributed to a prominent underlying mechanism of the anticancer potential of natural products (10). High specificity against cervical cancer cells along with minimizing the side effects on normal cells are considered as the main purpose of drug development strategies for treatment of cervical cancer (8). 

It has been demonstrated that oxidative stress is a prerequisite for promoting HPV-initiated cervical carcinogenesis and the potential cancer chemopreventive activities of natural antioxidants such as curcumin, ferulic acid, epigallocatechin-3-gallate (EGCG) and resveratrol can be promising toward cervical carcinoma treatment. Therefore, many oncological researchers are concentrating on novel therapeutic strategies based on natural products that counteract with oxidative stress. Previous studies have indicated that saponin metabolites show antioxidant efficacy that can be consider promising in reducing the danger of human cancers (11).

However, evidence-based researches showed saponin compounds derived from terrestrial sources possess anti cancer efficacy (12), which have been documented against cervical cancer. Thus, finding bioactive saponins from marine sources that selectively suppress cervical cancer proliferation without injury to normal tissue and prevent uncontrolled cancer cell division is critical for cervical cancer therapeutic approaches.

Recently we extracted and characterized the saponin fraction from the Persian Gulf brittle star *Ophiocoma erinaceus* Muller & Troschel (Ophiocomidae) with conventional extraction techniques (13).

 In his study, we explored the cytotoxicity of saponin fraction extracted from* O. erinaceus* in Hela-S3 cervical cancer and HNCF-PI52 cervical normal cell lines. In addition, the main focus of this study was the assessment of apoptosis inducing effect of brittle star saponin fraction against cervical cancer cells. 

## Experimental

*Chemicals*

Hela-S3 (cervical cancer cells) and HNCF-PI52 (normal cervical cells) were purchased from NCBI (National Cell Bank of Iran). DMEM Medium, FBS (Fetal Bovine Serum), trypsin-EDTA and antibiotic (Penicillin-streptomycin) were obtained from Gibco (USA). Specimens of the brittle star (*O. erinaceus*) were obtained from the rocky intertidal flats of the Persian Gulf. Methanol and ethanol were purchased from Merck (Germany). MTT (3-[4, 5- dimethyl thiazol-2-yl]-2,5-diphenyl tetrazolium bromide) and DAPI was prepared from AppliChem (USA). PI (propodium iodide) and acridine orange were obtained from Sigma (USA). Caspase-3 and caspase-9 colorimetric assay kits were purchased from Abcam (England). 

*Extraction and identification of crude saponin from brittle star*

Saponin fraction was extracted from the brittle star, *O. erinaceus* from the rocky intertidal flats of the Persian Gulf, the separation procedure was exerted on the basis of (14). Extraction method was performed using methanol, dichloromethane and *n*-butanol, sequentially. Isolation was performed using diaion HP-20, washed with dionized water, eluted with 80% and 100% ethanol. Subsequently, the 80% ethanol fraction was determined as saponin fraction and confirmation of saponin extraction was performed using TLC (Thin Layer Chromatography), erythrocyte lysis and FTIR (Fourier Transform Infrared spectroscopy) which was reported in our previous study (13).

*Cell Culture and cytotoxicity*

The Hela-S3 and HNCF-PI52 cells, were grown in DMEM medium containing 10% FBS supplemented with 1% antibiotic at 37 °C in a humidified, 5% CO_2_ incubator. The cells were maintained sub-confluent and all experiments were repeated at least three times. The inhibitory effects of brittle star saponin fraction on the growth of Hela-S3 cancer cells and HNCF-PI52 normal cell proliferation were measured by MTT assay. Briefly, the cells were cultivated at a concentration of 10^4^ cells/well in 96-well plates, overnight. Then the cells incubated with appropriate concentrations of brittle star saponin fraction (0, 1.5, 3.1, 6.25, 12.5, 25, 50, 100 µg/mL). The MTT assay was exerted 24 h and 48 h after treatment; the growth inhibitory effect of saponin fraction was determined by the addition of 10 µL MTT solution and dissolving formazan crystals with 80 µL DMSO, and, eventually, measurement of the optical absorbance of the dissolved formazan at 570 nm by spectrophotometer (Epoch, USA).

*DAPI staining*

In this assay, Hela-S3 cells were seeded on a coverslip in six well plates and then incubated with the different concentrations of brittle star saponin fraction for 24 h. Subsequently, the cells were fixed with methanol for 5 min at room temperature, rewashed and stained with 10 µg/mL DAPI at 37 °C for 15 min in the dark. Finally, morphological changes were observed under fluorescence microscope (Olympus, Japan).

*Apoptosis detection by Acridine Orange/Propodium Iodide (AO/PI)*

The cervical cancer cells (2 × 10^4^ cells/well) treated with a defined concentration of saponin fraction for 24 h were harvested and stained with 10 µL of a dye mixture comprising 100 µg/mL Acridine Orange (AO) and Propodium Iodide (PI). The cells were then mounted on coverslips and visualized under a fluorescence microscope for the morphological changes*.*

*Apoptosis analysis with Annexin V-FITC and propodium iodide method*

For evaluation of apoptosis induced by brittle star saponin fraction, the appearance of phosphatidylserine on the outer leaflet of membrane was evaluated. AnnexinV/propodium iodide staining was used with the Apoptosis Detection Kit (Abcam, UK) according to the manufacture instructions. Briefly, 24 h after treatment, Hela-S3 cells were trypsinized and resuspended in 500 µL of 1X binding buffer. Thereafter, 5 µL of Annexin-V-FITC and 5 µL of propodium iodide were added and incubated at room temperature for 5 min in the dark. In the next step, the samples were analyzed using FACSCalibur (Becton Dickinson, USA) flow cytometry.

*Flow cytometry analysis*

The cultivated Hela-S3 cells were exposed with a medium containing certain concentrations of brittle star saponin fraction. Then the cells were washed twice and resuspended in propodium iodide solution containing 0.1% sodium citrate plus 0.1% Triton X100 and incubated at 37 °C for 30 min, and then placed at 4 °C for 10 min and the fluorescence of stained cells was evaluated using a FACScan laser flow cytometer, and the population of cells was calculated using the WINMDI software.

*Colorimetric Caspase assay*

This assay was exerted using a colorimetric caspase-3 and -9 assay kit with quantification of caspase enzymatic activity according to cleavage of *p*-nitroaniline (pNA) from the labeled substrate DEVD-*p*NA and *p*-nitroanilide (*p*NA) from the labeled substrate LEHD-*p*-NA by active enzyme, respectively. Briefly, 4 × 10^6^ cells were exposed with appropriate concentrations of brittle star saponin fraction for 24 h. Then, the treated cells were trypsinized and lysed with 300 µL of chilled Cell Lysis Buffer, and centrifuged at 4°C to obtain supernatant cytosolic extract rich from protein content. Then, cell lysate were examined for measurement of caspase-3 and caspase-9 activities after the addition of 5 µL of 2X reaction buffer (containing dithioreitol) and 5 µL of the conjugated substrate and incubation at 37 °C for 2 h. Eventually, absorbance was read at 405 nm (Epoch, USA).

*Measurement of ROS generation*

Reactive oxygen species (ROS) production was determined by fluorescence microscopy and FACS using the method described previously with some modification. Briefly, after treatment, cells were incubated with (5μM2′-7′-dichloro-fluorescin diacetate) for 10 min. Cells were rinsed with PBS and then the intensity of DCFDA fluorescence determining the amount of intracellular ROS was analyzed using a flow cytometer with an excitation wavelength of 480 nm and an emission wavelength of 530 nm and evaluated under fluorescence microscope; photographs were taken.

*RNA extraction and RT-PCR analysis of Bcl-2*

In order to evaluate the effect of extracted saponin on Bcl-2 mRNA levels, 2×10^6^ Hela-S3 cells () were treated with different inhibitory concentrations of saponin. Then the total cellular RNA was isolated using the High Pure RNA Isolation kit (Roche, Germany). Total RNA (2 µg) was reverse-transcribed to cDNA using random hexamer, or oligodT, and RT premix; and then amplified using RT-PCR Premix (Pars Tous, Iran). First, the produced cDNA (2 µL) was added to 10 µL Taq premix, 2 µL forward primer, 2µL reverse primer and distilled water (Pars Tous, Iran). RT-PCR was performed 1 cycle reverse transcription at 95 °C/4 min and 35 cycles as denaturation at 94 °C for 30 s, annealing at 59 °C for 30 s, extension at 72 °C for 30 s and 1 cycles 5 min at 72 °C. The primers were: B2M Forward 5ꞌ TGGTGCTTGGCTCACTGACC 3ꞌꞌ , Reverse 5ꞌꞌ TATGTTCGGCTTCCCATTCT 3ꞌꞌ was used as the housekeeping gene. Forward 5ꞌꞌ CATGTGTGTGGAGAGCGTCAAC 3ꞌ and Reverse 5ꞌ CAGATAGGCACCCAGGGTGAT 3ꞌ for Bcl-2. The PCR products were electrophoresed on a 2% agarose gel and recorded using UV TEC gel documentation system (Cambridge, UK).

*Statistical analysis*

The results are presented as the mean ± SEM and experiments were carried out in triplicate. The difference between means was analyzed by one-way ANOVA followed by the Tukey test and Prism5. The level of p ≤ 0.05 was considered significant.

## Results and Discussion

In cancerous phenotype evasion of apoptosis and increment resistance to apoptosis results from up-regulation of anti-apoptotic members of Bcl-2 family (15). A variety of extracellular and intracellular stimuli can modulate mitochondrial mediated cell suicide including diverse physicochemical factors, anticancer compounds and free radical producing elements. Therefore, trying to find bioactive agents that elicit apoptosis (programmed cell death) is one purpose of cancer therapeutic modalities (16).

In respect of saponins, the high toxicity of compounds attributed to the low usefulness of compounds as a chemotherapeutic cytotoxic agents is one of the major drawbacks in drug discovery (17). Therefore, the identification of natural saponin with moderate toxicity on tumor cell lines may be useful in anticancer therapy (18). The permeabilzing properties of saponins have been proposed to be a prerequisite mechanism for the improvement drug delivery to enhance their cytotoxicity in addition to improved endocytosis (19). The reduction of metastasis, evocation of cell cycle arrest, inhibition of angiogenesis, prevention of drug efflux and elicit apoptosis are the most common causes of saponin application as an antitumor agent (20). 

**Figure 1:**
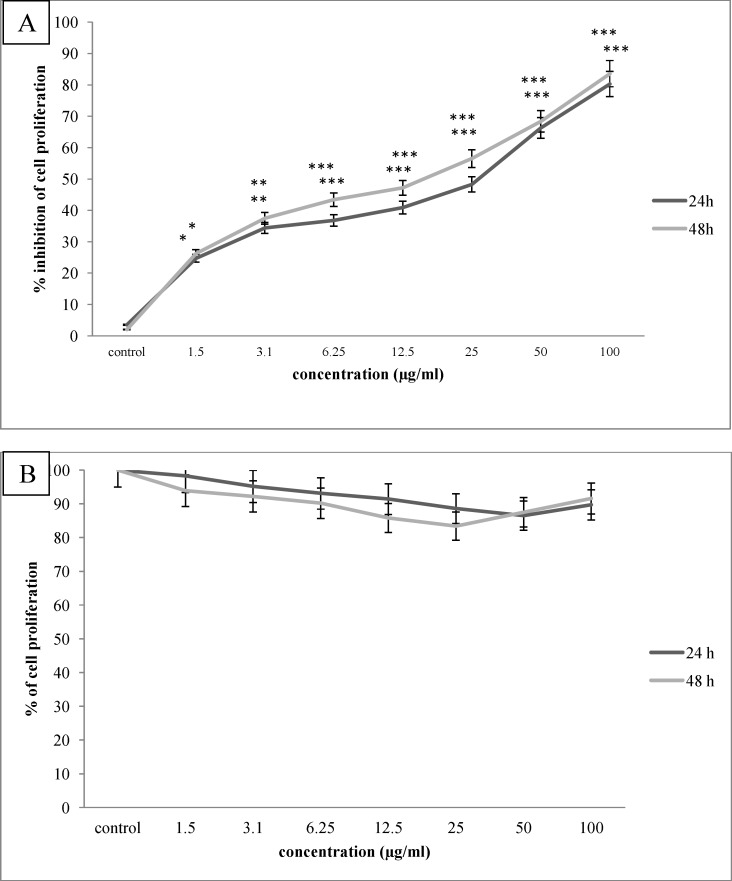
The anti proliferative effect of *O. erinaceus* saponin fraction on Hela-S3 cervical cancer cells (A) and HNCF-PI52 normal fibroblast cells (B) after 24,48 h incubation period. As indicated, saponin fraction inhibited cervical cell proliferation dose dependently, p value of *P<0.05, **P<0.01 and ***P<0.00, while did not inhibit normal cell proliferation, significantly

**Figure 2:**
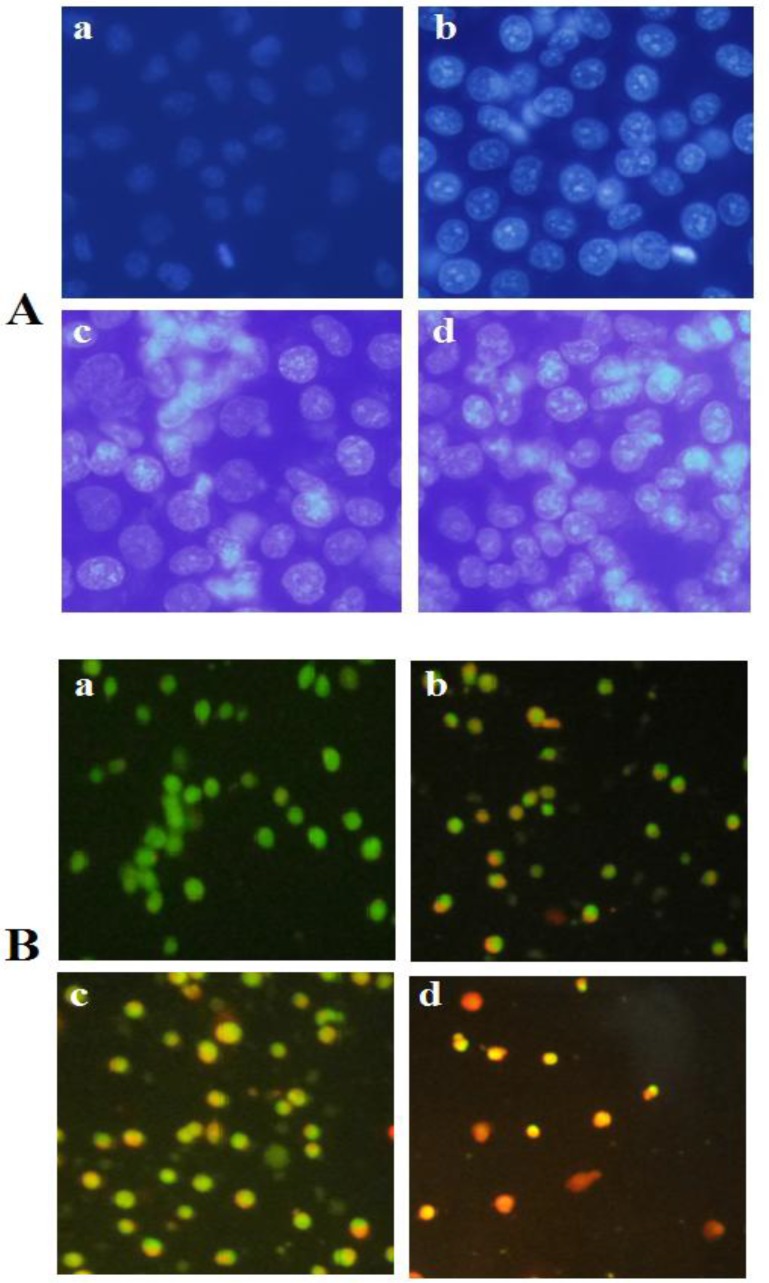
A) Fluorescence profiles of apoptosis induction by brittle star saponin fraction on cervical cancer cells using DAPI staining. (a-d) untreated cancer cells, treatment with 12.5, 25, 50 µg/ml brittle star saponin, respectively ×400. B) Fluorescence micrographs of brittle star saponin fraction on Hela-S3 cancer cells with AO/PI staining.(a-d) untreated cancer cells, treatment with 12.5, 25, 50 µg/ml brittle star saponin fraction. Magnification= ×200

**Figure 3:**
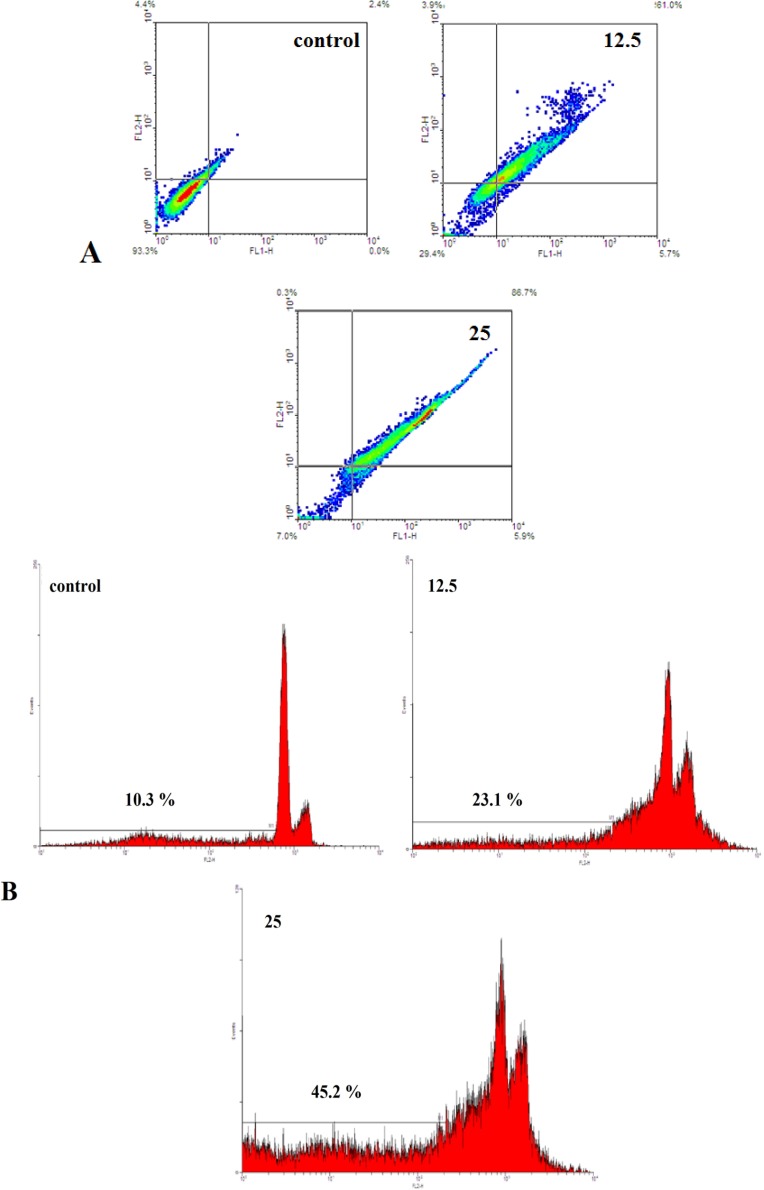
A) Evaluation of apoptosis by Annexin V-FITC method. The cells were exposed with increasing concentration of brittle star saponin fraction for 24 h by flow cytometry analysis.(a-d) untreated cancer cells, treatment with 12.5, 25 μg/ml brittle star saponin fraction, respectively. B) Apoptotic effect of brittle star saponin fraction on cervical cancer cells estimated by flow cytometry. Flow cytometry histogram of untreated and treated HeLa cells with 12.5, 25μg/ml brittle star saponin exhibited increase in sub-G1 region demonstrating mediation of an apoptotic cell death in cytotoxicity of brittle star saponin

**Figure 4:**
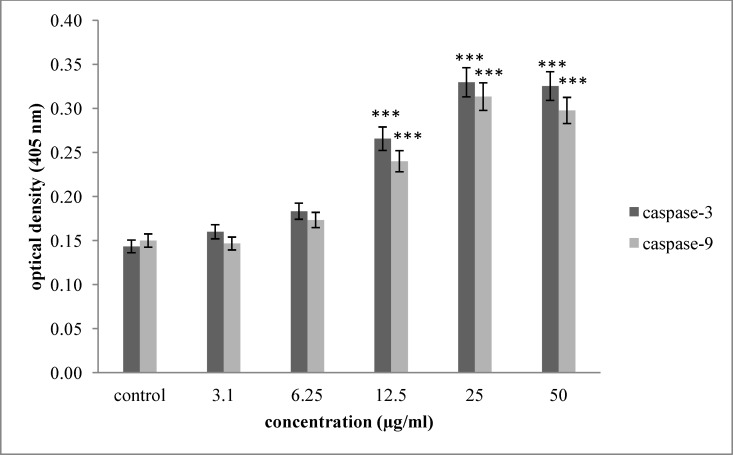
Effect of saponin fraction isolated from O.erinaceus on activation of caspase -3 and caspase -9. As shown, incubation of Hela-S3 cells with increasing concentration of brittle star saponin were enhanced significantly 24 h after treatment. P<0.05 were considered significant between experimental groups and control

**Figure 5:**
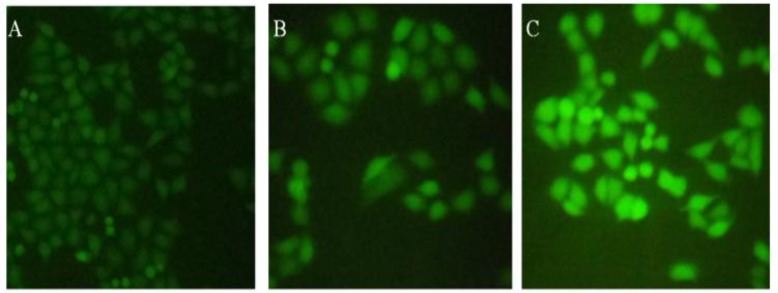
Effect of brittle star saponin fraction on intracellular ROS generation in Hela-S3 human cancer cells treated with brittle star saponin using fluorescence microscopy. A) untreated cells, B) 12.5 μg/ml, C) 25 μg/ml saponin treated cells. ×200

**Figure 6:**
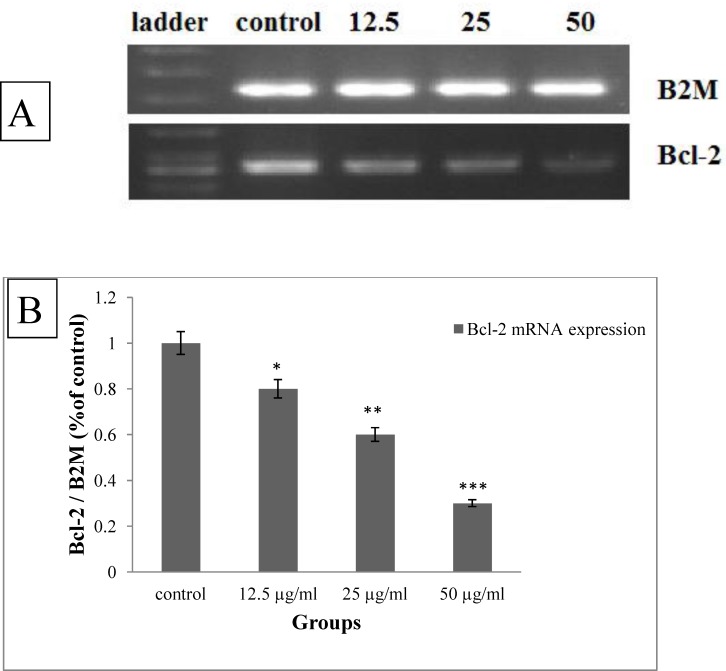
A) The qualitative changes in the expression of Bcl-2 mRNA under treatment with brittle star saponin by RT-PCR. B) The densitometry of gel quantitatively evaluated by image J (n = 3). *P<0.05, **P<0.01 and ***P<0.00 were considered significant

Although several investigations were conducted indicated that the anticancer effect of steroidal and terpenoid saponin (21), however, there is no documented literature referring to the anticancer potential of saponin fraction from Persian Gulf brittle star.

To determine the optimal concentrations of brittle star saponin fraction, the Hela-S3 cancer and HNCF-PI52 normal cells were treated with increasing concentrations (0, 3.1, 6.25, 12.5, 25, 50, 100 µg/mL) for 24 h and 48 h. Then the growth inhibitory effect was measured by MTT assay. As shown in Figure 1. the proliferation of Hela-S3 cancer cells was significantly inhibited in a dose dependent manner and the percentage of inhibition rate was 48.3%, 66.4% by 25 and 50 µg/mL after 24 h treatment (p ≤ 0.001) and 47.2%, 56.5% by 12.5 and 25 µg/mL after 48 h treatment(p ≤ 0.001), respectively. However, the HNCF-PI52 cells were less sensitive against exposure to brittle star saponin fraction, which indicated that treatment with brittle star saponin fraction administrated low toxicity against normal cells without a significant cytotoxic effect. The IC_50_ values of saponin fraction for Hela-S3 cells were considered approximately 23.4 µg/mL, which exhibited an inhibitory effect on cancer cells.

The results of DAPI staining exhibited chromatin fragmentation under treatment with definite concentrations of brittle star saponin fraction as one of characteristic of apoptosis cell death. In addition, AO/PI staining, through which the intact cells can be recognized by the green color; apoptotic cells are distinguished by the bright green or orange color; and necrotic cells by the red color. AO/PI staining revealed that exposure of Hela-S3 cancer cells to various concentrations of saponin induced apoptosis in IC_50_ value and necrosis cell death in the treatment with higher concentrations of saponin fraction; thus indicating apoptosis involvement in the cytotoxic effect of brittle star saponin on Hela-S3 cancer cells (Figure 2).

To verify the morphological changes under the treatment of Hela-S3 cancer cells with IC_50 _concentration of saponin fraction Annexin V-FITC/PI staining was performed. Flow cytometry diagrams (Figure 3A) determined the alive, early apoptotic, late apoptotic and necrotic cell death rate and demonstrated that with increasing concentration of saponin, the percentage of Annexin positive cells was enhanced remarkably. Hence, the percentage of apoptotic cells was gradually promoted from 2.4 % in the control group to 66.7 and 92.6% in the treated groups, which is indicative of the pro-apoptotic effect of the treatment with brittle star saponin fraction on Hela-S3 cervical tumor cells. 

The apoptosis inducing effect of saponin was assessed by flow cytometry analysis of propodium iodide according to the exclusion of propodium iodide (PI) by viable cells. As exhibited in Figure 3B, the treatment with saponin fraction significantly increased the sub-G1 peak, which means that the triggering of apoptosis is resulting from DNA fragmentation and endonuclease activation. Altogether, the results of apoptosis assays show that apoptosis is considered as a crucial anti proliferative mechanism of brittle star saponin fraction treatment.

In order to assess the mechanism of brittle star saponin fraction recruitment apoptosis, we examined the enzymatic activity of caspase-3 and caspase-9 colorimetrically. As exhibited in Figure 4. the activity of caspase-3, as apoptosis executioner enzyme, and caspase-9, as marker of intrinsic pathway, were significantly enhanced in a dose dependent manner with concentrations of 11.10 and 11.27 µg/mL (p ≤ 0.001) compared with the untreated cancer cells. Thus, increased activity of caspase-3 and caspase-9 shows the involvement of intrinsic pathway in apoptotic activity of brittle star saponin in Hela-S3 cervical cancer cells *in-vitro.*

To understand whether the pro apoptotic activity of brittle star saponin was attributed to the intracellular ROS levels of Hela-S3 cancer cells, the measurement of ROS level was performed via fluorochrome-based fluoroscopy with DCFH-DA. As indicated in Figure 5. the pretreatment of cancerous cells with brittle star saponin increased the fluorescence intensity compared with the control group, which proved that ROS production under treatment with brittle star saponin fraction was associated with the mitochondrial apoptotic pathway induced in cervical cancer cells.

To identify the type of induced pro-apoptotic activity of brittle star saponin, the cervical cancer cells were exposed to different concentrations of saponin and then RT-PCR assay was performed. Figure 6. 

shows dose dependently reduction of Bcl-2 mRNA expression (12.5, 25 µg/mL, p ≤ 0.05, 50 µg/mL, p ≤ 0.001). As, Bcl-2 family are involved in mitochondrial apoptotic pathway, therefore, the marked down-regulation Bcl-2 revealed that brittle star saponin exerted pro-apoptotic effect via intrinsic apoptotic pathway in cervical cancer cells.

Related with marine saponins, Cheng and colleagues (2006) examined the anticancer effect of asterosaponin1, a novel cytostatic compound from starfish, on human glioblastoma cells, and showed that this natural agent can significantly inhibit glioblastoma cell growth and induce apoptosis cell death with the up-regulation of Bax and down-regulation of Bcl-2. Thus, these results were proposed insights into the medical application of asterosaponin 1 in cancer chemotherapy (22). 

The survey on the antitumor potential of saponin extracted from sea cucumber showed that saponin separated from *Acaudina leucoprocta* revealed the highest toxicity toward S180 cancer cells with the IC_50_ concentration of 41.04 µg/mL indicating the anticancer potency of saponin extracted from marine organisms (23). There is a controversial survey and limited studies in the regard to the biological effect of saponin fraction from brittle star. Andersson and coworkers (1989) studied the biomedical activity of saponin fraction from brittle star and demonstrated that these compounds showed antibacterial activity against gram-positive bacterium and that only poly hydroxylated sterols indicated cytotoxicity against JURCAT and YAC-1 lymphoma cells *in-vitro*(24)*.*

## Conclusion

In summary, our observation elucidated that brittle star saponin fraction have a marked growth inhibitory effect on Hela-S3 cervical cancer, while indicating a less cytotoxic effect on HNCF-PI52 normal cells. In addition, the anticancer proliferative activity of brittle star saponin fraction is associated with triggering apoptosis cell death, which was proven by fluorescence microscopy, Annexin V-FITC staining and flow cytometry. Moreover, the increment of ROS intracellular level indicated counteracting saponin isolated with stress oxidative in cervical cancer cells. The activation of caspase-3, caspase-9 and down regulation of Bcl-2 mRNA demonstrated mitochondrial mediated apoptosis in cervical cancer cells, thus delineating brittle star saponin fraction as potent anticancer agents in the treatment of cervical cancer. 

## Conflict of interest

There is no conflict of interest.
